# Dichlorobenzene: an effective solvent for epoxy/graphene nanocomposites preparation

**DOI:** 10.1098/rsos.170778

**Published:** 2017-10-11

**Authors:** Jiacheng Wei, Mohd Shahneel Saharudin, Thuc Vo, Fawad Inam

**Affiliations:** Department of Mechanical and Construction Engineering, Northumbria University, Newcastle upon Tyne NE1 8ST, UK

**Keywords:** epoxy, graphene, nanocomposites, dichlorobenzene, solvent

## Abstract

It is generally recognized that dimethylformamide (DMF) and ethanol are good media to uniformly disperse graphene, and therefore have been used widely in the preparation of epoxy/graphene nanocomposites. However, as a solvent to disperse graphene, dichlorobenzene (DCB) has not been fully realized by the polymer community. Owing to high values of the dispersion component (*δ*_d_) of the Hildebrand solubility parameter, DCB is considered as a suitable solvent for homogeneous graphene dispersion. Therefore, epoxy/graphene nanocomposites have been prepared for the first time with DCB as a dispersant; DMF and ethanol have been chosen as the reference. The colloidal stability, mechanical properties, thermogravimetric analysis, dynamic mechanical analysis and scanning electron microscopic images of nanocomposites have been obtained. The results show that with the use of DCB, the tensile strength of graphene has been improved from 64.46 to 69.32 MPa, and its flexural strength has been increased from 97.17 to 104.77 MPa. DCB is found to be more effective than DMF and ethanol for making stable and homogeneous graphene dispersion and composites.

## 1. Introduction

Epoxy resins can be seen to be widely used in aerospace, automotive, marine, structures and construction, owing to their superlative mechanical properties and favourable thermal stability, which are important for various applications [1,2]. Epoxy resins are of particular interest to engineering because these resins provide a unique balance of chemical and mechanical properties combined with ease of processing [3].

For epoxy/graphene nanocomposites, graphene can significantly improve the physical and chemical properties of matrix at extremely low loadings, and this improvement could only be achieved when the filler is homogeneously dispersed in the matrix [4,5]. The uniformly dispersed graphene could share external stress and blocks advancing cracks, which improve the mechanical properties [6–8]. However, for practical applications, graphene is not suitable to disperse in epoxy just by simple mixing, as graphene tends to reaggregate in the matrix due to the strong van der Waals force between separately dispersed graphene sheets [9,10].

Therefore, solvents are widely used as dispersants to overcome the van der Waals force between graphene nanosheets, and hence lead to uniform graphene dispersion. For example, ethanol [11–14] has been widely adopted as a dispersant for graphene materials, and showed good dispersability and stability. Dimethylformamide (DMF) [15–20] is also well recognized in polymer communities as a good dispersant for graphene. By using DMF, a lot of works reported enhancements in the final properties of epoxy/graphene nanocomposites. Other solvents like tetrahydrofuran [21–24], acetone [25–28], dichloromethane [29–32], isopropyl alcohol [33,34], water [35–39], etc. have also been reported in the processing of epoxy/graphene nanocomposites.

Besides those commonly used solvents, dichlorobenzene (DCB) has also been widely reported as a good dispersant for preparing graphene dispersions because of the following reasons. Firstly, DCB is a commonly used reaction solvent for fullerenes and is known to form stable single-walled nanotube dispersions [40]. Secondly, DCB is a convenient dispersant and is compatible with a variety of chemicals. Thirdly, DCB, being aromatic, is able to interact with graphene via π–π stacking [41]. Fourthly, it has been reported that solvents with high values of the dispersion component (*δ*_d_) of the Hildebrand solubility parameter are the best for making homogeneous and aggregate-free dispersions of graphene [42,43]. DCB shows a high *δ*_d_ of 19.2 MPa^1/2^. In the light of this, DCB tends to be suitable to produce stable graphene dispersion. Song *et al*. [44] dispersed graphene in DCB, and tested the stability of the dispersion by measuring its light absorbance. Naebe *et al*. [45] surface-treated graphene with DCB to prepare functionalized graphene. Some other works [46–53] also involved the use of DCB and reported positive effects.

However, though DCB has been used a lot in the dispersion of graphene, the use of DCB to prepare epoxy/graphene nanocomposites is not yet fully realized by the polymer community. To the best of our knowledge, there is no work reported using DCB as a dispersant for epoxy/graphene nanocomposites. In this work, *o*-DCB has been chosen and used for the first time to prepare epoxy/graphene nanocomposites. Another two commonly used solvents, DMF (*δ*_d_ = 17.4 MPa^1/2^) and ethanol (*δ*_d_ = 15.8 MPa^1/2^), have been used for comparison in this work.

## 2. Material and methods

### 2.1. Materials

The epoxy system used in this study was purchased from Polyfibre UK Ltd. This system offers good all-round properties and consists of a EPOPHEN EL5 bisphenol A-based liquid epoxy and EPOPHENEHA 57 diamine hardener. Graphene nanoplatelets were offered by Graphene Laboratories Inc., USA with an average lateral size of 4.5 µm and a specific surface area of 80 m^2^ g^−1^. *o*-DCB, DMF and ethanol were purchased from Sigma-Aldrich Company with a purity of 99.9%.

### 2.2. Sample preparation

According to our previous research [7], 0.3 wt% graphene loading shows the maximum property enhancement to epoxy; therefore, in this work, 0.3 wt% nanocomposites were prepared with different solvents to compare their dispersing efficiencies.

One set of samples was prepared without any solvent, marked as G-0.3. DCB, DMF and ethanol were used to prepare another three sets of samples; 0.45 g of graphene was first dispersed in 100 ml of solvent (DCB, DMF and ethanol, respectively) and bath-sonicated for 0.5 h; then 99.7 g of epoxy monomer was added to the dispersion and sonicated for another 0.5 h. To remove the solvent, the mixtures were heated with stirring; all the mixtures were then weighed to ensure evaporation of the solvents fully. Then the mixtures were cooled down to room temperature and 49.85 g of hardener was added with hand stirring for 5 min followed by 5 min of bath sonication; then the entrapped air bubbles were removed by vacuum degassing. Finally, the liquid mixtures were mould cast and cured for 6 h at room temperature and then for 6 h at 80°C.

### 2.3. Characterization

Tensile properties, flexural properties and fracture toughness were measured by a universal mechanical testing machine (Instron 3382); for all tests the crosshead speed used was 2 mm min^−1^. Tensile tests were conducted according to ASTMD638 (Type V) with a specimen thickness of 4 mm. Flexural properties were measured according to ASTM D790 with a specimen dimension of 48 × 12.7 × 3 mm. A single-edge-notch three-point bend specimen was used to measure the fracture toughness (*K*_1C_) according to ASTM D5045; the specimen dimension was 36 × 6 × 3 mm with a crack of length of 3 mm. The *K*_1C_ was calculated using

2.1 $$K_{1C}=\frac{P_{\max}f(a\text{/}w)}{BW^{1/2}},$$

where *W* is the sample width (millimetres), *a* is the crack length and kept between 0.45 and 0.55 *W*, *B* is the sample thickness (millimetres), *f*(*a*/*w*) was calculated using equation (2.2), which is constant and related to the sample geometry and *P*_max_ is the maximum load of the load–displacement curve. The critical strain energy release rate (*G*_1C_) was calculated using equation (2.3), where *v* is Poisson's ratio of the polymer and taken as 0.35 and *E* is Young's modulus obtained from the tensile tests (MPa).

2.2 $$f\left(\frac{a}{w}\right)=\frac{\left(2+a\text{/}w)\{0.0866+4.64(a\text{/}w)-13.32{\left(a\text{/}w\right)}^{2}+14.72{\left(a\text{/}w\right)}^{3}-5.6{\left(a\text{/}w\right)}^{4}\right\}}{{\left(1-a\text{/}w\right)}^{3/2}}$$

and

2.3 $$G_{1C}=\frac{K_{1C}^{2}(1-v^{2})}{E}.$$

Vickers microhardness was measured by Buehler Micromet II. A dwell time of 10 s with a load of 200 g was used for all the samples. Six samples were measured for each set of conditions and mean values were then reported.

A Perkin Elmer-8000 dynamic mechanical analysis (DMA) instrument was used to conduct the analysis to determine the storage modulus (*E*′) and loss factor (tan *δ*). Sample dimensions of 30 × 8 × 2.5 mm were tested by the single cantilever method. All tests were carried out under the temperature sweeping mode (temperature ramp from 30°C to 150°C at 5°C min^−1^) at a constant frequency of 1 Hz. The glass transition temperature (*T*_g_) was taken as the temperature value at the peak of tan *δ* curves. Thermogravimetric analysis (TGA) of the nanocomposites was carried out with a TA Instruments Q500 thermal analyser. The temperature range was from room temperature to 600°C at a ramp rate of 5°C min^−1^ under N_2_ atmosphere. To evaluate the fracture modes of the nanocomposites, a FEI Quanta 200 electron microscope was used to obtain scanning electron microscopy (SEM) images. A layer of gold with 10 nm thickness was applied to the samples' fractured surfaces using an Emscope sputter coater model SC500A.

## 3. Results and discussion

### 3.1. Visual stability

**Figure 1.**
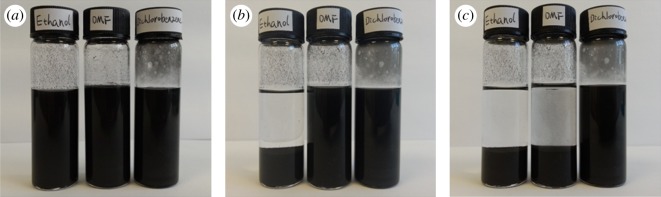
Visual stability of 3 g l^−1^ graphene suspensions (*a*) 5 min after sonication, (*b*) 2 h after sonication and (*c*) 12 h after sonication. Samples from left to right: ethanol, DMF, DCB.

**Figure 2.**
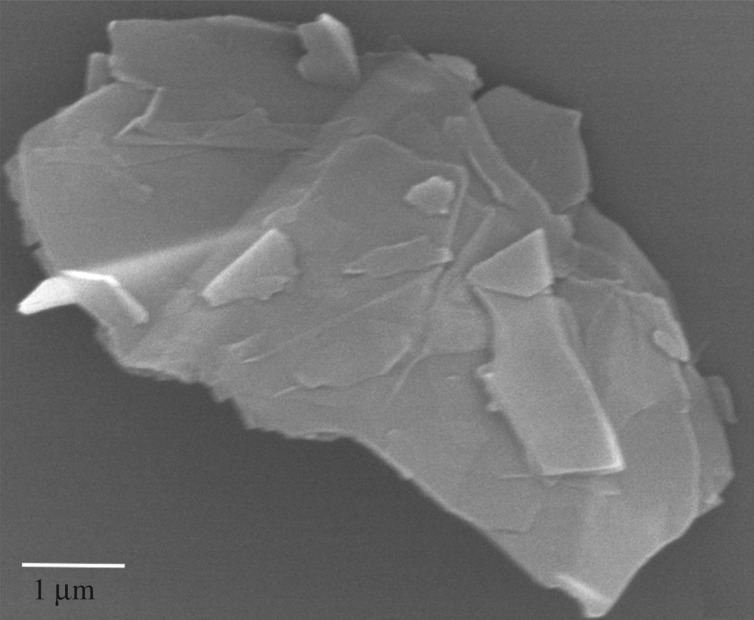
SEM image of as-received multilayer graphene.

Successful fabrication of nanocomposites crucially depends on maintaining a stable dispersion of graphene before polymer curing. Figure 1 shows the colloidal suspensions of graphene in DCB, DMF and ethanol at different time intervals after sonication. The pictures show that graphene settled down in ethanol within two hours after sonication; the graphene–DMF suspension also reaggregated significantly and settled down within 12 h of sonication. However, stable dispersion was achieved only by DCB, which suggests that DCB is the best for preparing uniformly dispersed epoxy/graphene nanocomposites. Figure 2 shows the SEM image of as-received stacked multilayer graphene.

### 3.2. Mechanical properties

**Figure 3.**
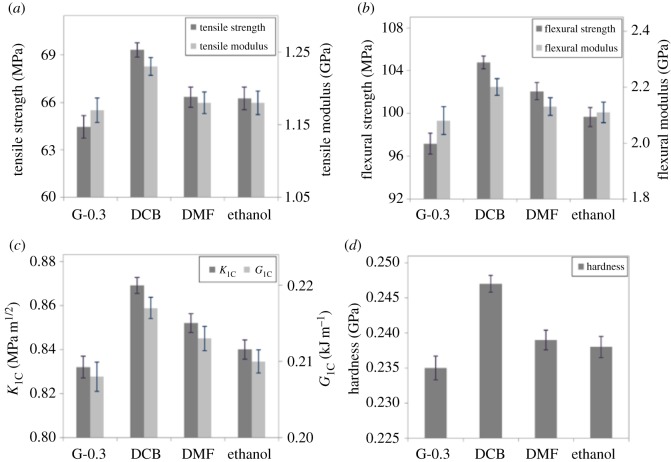
Mechanical properties of epoxy/graphene nanocomposites: (*a*) tensile properties, (*b*) flexural properties, (*c*) fracture properties and (*d*) hardness.

The uniformly dispersed graphene in the matrix will in return affect the macroscopic properties of the nanocomposites, while most of the work on epoxy/graphene nanocomposites aims at exploiting the mechanical enhancement of the nanocomposites. Therefore, the mechanical properties of nanocmposites have been tested and are summarized in figure 3.

As can be seen from the figure, G-0.3 shows the lowest values in the properties, such as a tensile strength of 64.46 MPa, flexural strength of 97.17 MPa and hardness of 0.235 GPa. DCB samples show the highest properties, with a tensile strength of 69.32 MPa, flexural strength of 104.77 MPa and Vickers hardness of 0.247 GPa. Meanwhile, DMF- and ethanol-prepared samples show only medium increase in the properties, indicating that DCB produces better graphene dispersion than DMF and ethanol. To sum up, the improvements in the macroscopic properties of epoxy/graphene nanocomposites are due to the enhanced dispersion of graphene. The uniformly dispersed graphene forms a continuous network in the matrix, which could release the stress concentration, and thus enhance the mechanical strength and improve the energy-absorbing capacity. Moreover, the uniformly dispersed graphene has changed the microstructure of the polymeric network, which will be discussed in below.

### 3.3. Thermogravimetric analysis test

**Figure 4.**
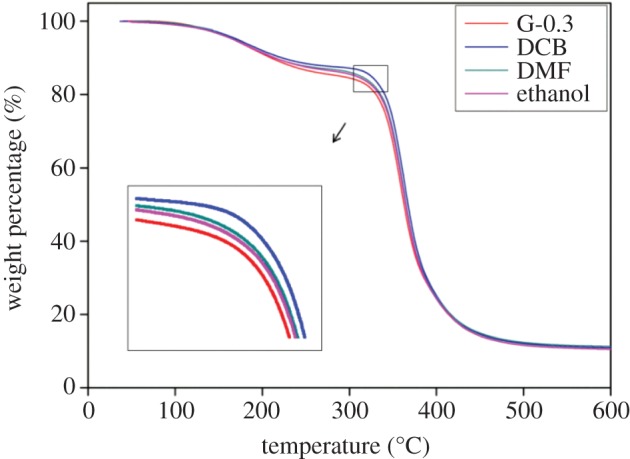
TGA curves of the nanocomposites.

Thermal decomposition is one of the fundamental thermal properties and is critical for practical applications. Figure 4 shows the TGA curves of the nanocomposites in a nitrogen atmosphere. It can be seen that all samples have a similar two-stage weight loss, indicating that all the samples have a similar thermal degradation mechanism. The first weight loss from 100°C to 230°C was attributed to the decomposition of small molecules on the side chain. The second weight loss that occurred from 250°C to 500°C shows the decomposition of the main polymer chain [54,55]. As can be seen from the figure, DCB samples show the lowest decomposition rate, which means the highest thermal stability compared with that of the DMF and ethanol samples.

The reason of this phenomenon can be explained by the fact that graphene has affected the cross-linking structure of the matrix. Generally, the cross-linking density means the concentration of cross-linked bonds per volume. For typical polymeric materials, the higher the cross-linking density, the stronger the polymer chains bond with each other, thus resulting in a stronger capacity to withstand heat. Compared to the structures of DMF- and ethanol-prepared samples, DCB-prepared samples tend to produce more uniform graphene dispersion, and thus an increase in the cross-linking density [56,57]. On the other hand, the homogeneously dispersed graphene could form a continuous network in the matrix, and thus could reduce the volatilization rate of the decomposition products.

In general, the increased thermal stability of DCB samples resulted in a higher heat capacity of nanocomposites and a better barrier effect of the graphene network, which was caused by the uniform dispersion of graphene by using DCB.

### 3.4. Dynamic mechanical analysis test

**Figure 5.**
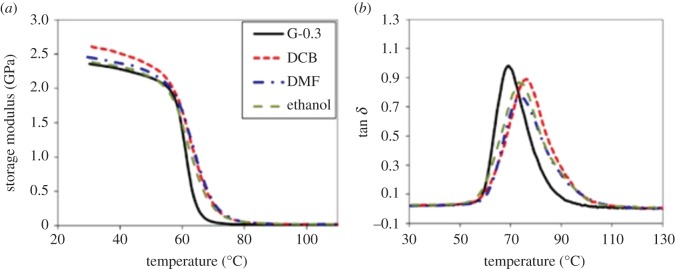
DMA curves of nanocomposites: (*a*) storage modulus and (*b*) tan *δ.*

Figure 5*a* shows the storage moduli (*E*′) of the nanocomposites. As can be seen from the figure, the storage moduli of samples prepared with solvents increased obviously over the G-0.3 samples throughout the temperature range. Especially, DCB-prepared samples show a 2.61 GPa storage modulus, which is higher than the 2.45 GPa of DMF samples and 2.38 GPa of ethanol samples.

The segmental motion of polymers is characterized by glass transition temperature (*T*_g_), which was taken as the temperature value at the peak of tan *δ* curves and are shown in figure 5*b*. In this figure, it can be seen that the tan *δ* peak is observed at 69.28°C for G-0.3 samples. For nanocomposites processed by DCB, DMF and ethanol, the *T*_g_ were observed at higher temperatures. This can be ascribed to the phenomenon that the uniformly dispersed graphene has restricted the chain mobility of the epoxy matrix, and thus increased the *T*_g_ values [58]. Among all the samples, DCB-prepared samples show the highest *T*_g_ of 76.57°C, which is a more than 7°C increment compared with the G-0.3 samples, while only slight increases (approx. 4°C) in *T*_g_ are obtained for samples prepared with DMF and ethanol. As mentioned above, the uniformly dispersed graphene increased the cross-linking density of the nanocomposites, and then plays a positive role in improving the thermal stability.

### 3.5. Scanning electron microscopy test

**Figure 6.**
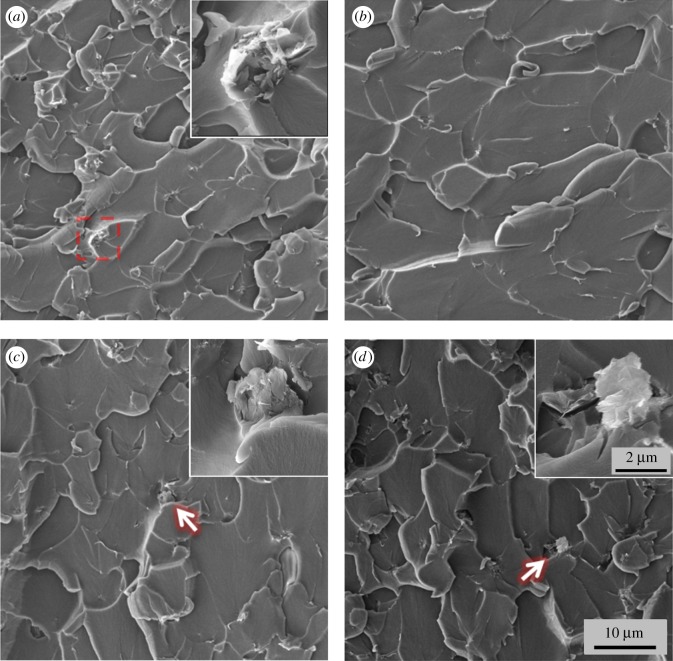
SEM images of fracture surfaces of (*a*) G-0.3, (*b*) DCB samples, (*c*) DMF samples and (*d*) ethanol samples.

The fracture surfaces of nanocomposites were examined by SEM and are shown in figure 6. For G-0.3 samples, as shown in figure 6*a*, graphene aggregates are sparsely located on the surface; the inset of figure 6*a* shows the typical morphology of a graphene aggregate. This poorly dispersed surface indicates a poor interfacial interaction between the epoxy matrix and graphene, and features the brittle nature of the material and its poor resistance to crack initiation and propagation. Compared to the G-0.3 samples, the fracture surfaces of DCB samples are smoother, as shown in figure 6*b*. The clear fracture patterns show the fracture mechanism of sheet/sheet delamination of this nanocomposite, and reveals that the usage of DCB leads to uniform graphene dispersion. The homogeneously dispersed graphene is able to bridge growing cracks, impede crack propagation and thus improve the properties of nanomaterials. However, for DMF- and ethanol-prepared samples, as shown by the insets in figure 6*c*,*d*, some poorly dispersed graphene can still be seen on the surface. The non-uniformly dispersed graphene forms defects in the nanocomposites, which act to concentrate the stresses locally, effectively causing a localized weakness, thus decreasing the properties of the nanocomposites.

## 4. Conclusion

A prerequisite for property enhancement of epoxy/graphene nanocomposites is the uniform dispersion of graphene in the matrix; however, the ultra-high specific surface area of graphene results in high van der Waals forces and thus induces a strong tendency to reaggregate. Solvents are able to provide a low-viscous medium, which is an ideal environment for graphene to disperse. While dispersing, lower viscosities are responsible for promoting the de-bundling of graphene sheets. This results in the triggering of the van der Waals force on graphene surfaces in lower proximity with each other, thus leading to dispersion. Therefore, the selection of dispersion media is very important for the final properties of nanocomposites.

In this work, DCB was selected to test its effectiveness for epoxy/graphene nanocomposites preparation. The colloidal stability and mechanical properties were determined, and TGA, DMA and SEM imaging of nanocomposites were conducted. The results show that DCB is able to produce stable graphene dispersion; however, DMF and ethanol are not sufficient to disperse graphene homogeneously in the nanocomposites. Nanocomposites prepared with DCB also show higher mechanical properties and better thermal stability compared to the samples prepared with DMF and ethanol.

In general, it is concluded that DCB is more efficient than DMF and ethanol for making uniform and stable graphene dispersions. The usage of DCB can endow the nanocomposites with outstanding mechanical properties and improved thermal stability. This finding can be significant in practical applications and gives an impetus for DCB usage not only in epoxy/graphene nanocomposites preparation, but also in other polymer nanocomposites where the usage of solvents is required in the processing.

## Data accessibility

All the data are provided in the main text.

## References

[1] Hodgkin JH, Simon GP, Varley RJ 1998 Thermoplastic toughening of epoxy resins: a critical review. Polym. Adv. Technol. 9, 3–10. (doi:10.1002/(SICI)1099-1581(199801)9:1<3::AID-PAT727>3.0.CO;2-I)

[2] Wei J, Vo T, Inam F 2015 Epoxy/graphene nanocomposites—processing and properties: a review. RSC Adv. 5, 73 510–73 524. (doi:10.1039/C5RA13897C)

[3] King JA, Klimek DR, Miskioglu I, Odegard GM 2014 Mechanical properties of graphene nanoplatelet/epoxy composites. J. Compos. Mater. 49, 659–668. (doi:10.1002/app.38645)

[4] Kumar R, Singh RK, Singh DP, Joanni E, Yadav RM, Moshkalev SA 2017 Laser-assisted synthesis, reduction and micro-patterning of graphene: recent progress and applications. Coord. Chem. Rev. 342, 34–79. (doi:10.1016/j.ccr.2017.03.021)

[5] Awasthi S, Awasthi K, Kumar R, Srivastava ON 2009 Functionalization effects on the electrical properties of multi-walled carbon nanotube-polyacrylamide composites. J. Nanosci. Nanotechnol. 9, 5455–5460. (doi:10.1166/jnn.2009.1160) 19928243 10.1166/jnn.2009.1160

[6] Galpaya D, Wang M, George G, Motta N, Waclawik E, Yan C 2014 Preparation of graphene oxide/epoxy nanocomposites with significantly improved mechanical properties. J. Appl. Phys. 116, 053518 (doi:10.1063/1.4892089)

[7] Wei J, Atif R, Vo T, Inam F 2015 Graphene nanoplatelets in epoxy system: dispersion, reaggregation, and mechanical properties of nanocomposites. J. Nanomater. 2015, 561742 (doi:10.1155/2015/561742)

[8] Li Z, Young RJ, Wang R, Yang F, Hao L, Jiao W, Liu W 2013 The role of functional groups on graphene oxide in epoxy nanocomposites. Polymer 54, 5821–5829. (doi:10.1016/j.polymer.2013.08.026)

[9] Paredes JI, Villar-Rodil S, Martinez-Alonso A, Tascon JMD 2008 Graphene oxide dispersions in organic solvents. Langmuir 24, 10 560–10 564. (doi:10.1021/la801744a) 10.1021/la801744a 18759411

[10] Mittal V 2014 Functional polymer nanocomposites with graphene: a review. Macromol. Mater. Eng. 299, 906–931. (doi:10.1002/mame.201300394)

[11] Zeng C, Lu S, Xiao X, Gao J, Pan L, He Z, Yu J 2014 Enhanced thermal and mechanical properties of epoxy composites by mixing noncovalently functionalized graphene sheets. Polym. Bull. 72, 453–472. (doi:10.1007/s00289-014-1280-5)

[12] Kumar R, Singh RK, Singh DP, Savu R, Moshkalev SA 2016 Microwave heating time dependent synthesis of various dimensional graphene oxide supported hierarchical ZnO nanostructures and its photoluminescence studies. Mater. Design 111, 291–300. (doi:10.1016/j.matdes.2016.09.018)

[13] Qiao SJ, Xu XN, Qiu Y, Xiao HC, Zhu YF 2016 Simultaneous reduction and functionalization of graphene oxide by 4-hydrazinobenzenesulfonic acid for polymer nanocomposites. Nanomaterials 6, 29 (doi:10.3390/nano6020029) 10.3390/nano6020029 PMC5302486 28344286

[14] Li W, Wang M, Yue Y, Ji W, Ren R 2016 Enhanced mechanical and thermal properties of bismaleimide composites with covalent functionalized graphene oxide. RSC Adv. 6, 54 410–54 417. (doi:10.1039/C6RA09260H)

[15] Monfared Zanjani JS, Okan BS, Menceloglu YZ, Yildiz M 2016 Nano-engineered design and manufacturing of high-performance epoxy matrix composites with carbon fiber/selectively integrated graphene as multi-scale reinforcements. RSC Adv. 6, 9495–9506. (doi:10.1039/C5RA23665G)

[16] Wang J, Wang J, Xu R, Sun Y, Zhang B, Chen W, Wang T, Yang S 2015 Enhanced microwave absorption properties of epoxy composites reinforced with Fe_50_Ni_50_-functionalized graphene. J. Alloys Compd. 653, 14–21. (doi:10.1016/j.jallcom.2015.08.278)

[17] Wang J, Sun Y, Chen W, Wang T, Xu R, Wang J 2015 Enhanced microwave absorption performance of lightweight absorber based on reduced graphene oxide and Ag-coated hollow glass spheres/epoxy composite. J. Appl. Phys. 117, 154903 (doi:10.1063/1.4917486)

[18] Kumar R, Singh RK, Vaz AR, Savu R, Moshkalev SA 2017 Self-assembled and one-step synthesis of interconnected 3D network of Fe_3_O_4_/reduced graphene oxide nanosheets hybrid for high-performance supercapacitor electrode. ACS Appl. Mater. Interfaces 10, 8880–8890. (doi:10.1021/acsami.6b14704) 10.1021/acsami.6b14704 28225588

[19] Wang J, Wang J, Zhang B, Sun Y, Chen W, Wang T 2016 Combined use of lightweight magnetic Fe_3_O_4_-coated hollow glass spheres and electrically conductive reduced graphene oxide in an epoxy matrix for microwave absorption. J. Magn. Magn. Mater. 401, 209–216. (doi:10.1016/j.jmmm.2015.10.001)

[20] Zha JW, Zhang B, Li RKY, Dang ZM 2016 High-performance strain sensors based on functionalized graphene nanoplates for damage monitoring. Compos. Sci. Technol. 123, 32–38. (doi:10.1016/j.compscitech.2015.11.028)

[21] Ma Q, Luo J, Chen Y, Wei W, Liu R, Liu X 2015 Reactive copolymer functionalized graphene sheet for enhanced mechanical and thermal properties of epoxy composites. J. Polym. Sci. A Polym. Chem. 53, 2776–2785. (doi:10.1002/pola.27751)

[22] Zaman I, Manshoor B, Khalid A, Meng Q, Araby S 2014 Interface modification of clay and graphene platelets reinforced epoxy nanocomposites: a comparative study. J. Mater. Sci. 49, 5856–5865. (doi:10.1007/s10853-014-8296-y)

[23] Meng Q, Jin J, Wang R, Kuan HC, Ma J, Kawashima N, Michelmore A, Zhu S, Wang CH 2014 Processable 3-nm thick graphene platelets of high electrical conductivity and their epoxy composites. Nanotechnology 25, 125707 (doi:10.1088/0957-4484/25/12/125707) 24577240 10.1088/0957-4484/25/12/125707

[24] Wang Z, Wei P, Qian Y, Liu J 2014 The synthesis of a novel graphene-based inorganic–organic hybrid flame retardant and its application in epoxy resin. Compos. B Eng. 60, 341–349. (doi:10.1016/j.compositesb.2013.12.033)

[25] Liu X, Sun X, Wang Z, Shen X, Wu Y, Kim JK 2015 Planar porous graphene woven fabric/epoxy composites with exceptional electrical, mechanical properties, and fracture toughness. ACS Appl. Mater. Interfaces 7, 21 455–21 464. (doi:10.1021/acsami.5b06476) 10.1021/acsami.5b06476 26331902

[26] Wan YJ, Yang WH, Yu SH, Sun R, Wong CP, Liao WH 2016 Covalent polymer functionalization of graphene for improved dielectric properties and thermal stability of epoxy composites. Compos. Sci. Technol. 122, 27–35. (doi:10.1016/j.compscitech.2015.11.005)

[27] Singh RK, Kumar R, Singh DP 2016 Graphene oxide: strategies for synthesis, reduction and frontier applications. RSC Adv. 69, 64 993–65 011. (doi:10.1039/C6RA07626B)

[28] Kumar R, Singh RK, Singh DP 2016 Natural and waste hydrocarbon precursors for the synthesis of carbon based nanomaterials: graphene and CNTs. Renew. Sustain. Energ. Rev. 58, 976–1006. (doi:10.1016/j.rser.2015.12.120)

[29] Liu T, Zhao Z, Tjiu WW, Lv J, Wei C 2014 Preparation and characterization of epoxy nanocomposites containing surface-modified graphene oxide. J. Appl. Polym. Sci. 131, 40236 (doi:10.1002/app.40236)

[30] Cao L, Liu X, Na H, Wu Y, Zheng W, Zhu J 2013 How a bio-based epoxy monomer enhanced the properties of diglycidyl ether of bisphenol A (DGEBA)/graphene composites. J. Mater. Chem. A 1, 5081–5088. (doi:10.1039/C3TA01700A)

[31] Yu G, Wu P 2014 Effect of chemically modified graphene oxide on the phase separation behaviour and properties of an epoxy/polyetherimide binary system. Polym. Chem. 5, 96–104. (doi:10.1039/C3PY00878A)

[32] Hou G, Gao J, Xie J, Li B 2016 Preparation and properties characterization of gallic acid epoxy resin/succinic anhydride bionanocomposites modified by green reduced graphene oxide. Soft Mater. 14, 27–37. (doi:10.1080/1539445X.2015.1098704)

[33] Barletta M, Vesco S, Puopolo M, Tagliaferri V 2016 Graphene reinforced UV-curable epoxy resins: design, manufacture and material performance. Prog. Org. Coat. 90, 414–424. (doi:10.1016/j.porgcoat.2015.08.013)

[34] Barletta M, Vesco S, Puopolo M, Tagliaferri V 2015 High performance composite coatings on plastics: UV-curable cycloaliphatic epoxy resins reinforced by graphene or graphene derivatives. Surf. Coat. Technol. 272, 322–336. (doi:10.1016/j.surfcoat.2015.03.046)

[35] Wang C, Ge H, Liu H, Guo S 2015 Microstructure and properties of carbon fiber sized with Pickering emulsion based on graphene oxide sheets and its composite with epoxy resin. J. Appl. Polym. Sci. 132, 42285 (doi:10.1002/app.42285)

[36] Nasab MG, Kalaee M 2016 Epoxy/graphene oxide/liquid polysulfide ternary nano-composites: rheological, thermal and mechanical, characterization. RSC Adv. 6, 45 357–45 368. (doi:10.1039/C6RA05919H)

[37] Ramezanzadeh B, Niroumandrad S, Ahmadi A, Mahdavian M, Moghadam MHM 2016 Enhancement of barrier and corrosion protection performance of an epoxy coating through wet transfer of amino functionalized graphene oxide. Corros. Sci. 103, 283–304. (doi:10.1016/j.corsci.2015.11.033)

[38] Ming P, Zhang Y, Bao J, Liu G, Li Z, Jiang L, Cheng Q 2015 Bioinspired highly electrically conductive graphene–epoxy layered composites. RSC Adv. 5, 22 283–22 288. (doi:10.1039/C5RA00233H)

[39] Wang Z, Shen X, Akbari Garakani M, Lin X, Wu Y, Liu X, Sun X, Kim JK 2015 Graphene aerogel/epoxy composites with exceptional anisotropic structure and properties. ACS Appl. Mater. Interfaces 7, 5538–5549. (doi:10.1021/acsami.5b00146) 25691257 10.1021/acsami.5b00146

[40] Bahr JL, Mickelson ET, Bronikowski MJ, Smalley RE, Tour JM 2001 Dissolution of small diameter single-wall carbon nanotubes in organic solvents. Chem. Commun. 193–194. (doi:10.1039/B008042J)

[41] Hamilton CE, Lomeda JR, Sun Z, Tour JM, Barron AR 2009 High-yield organic dispersions of unfunctionalized graphene. Nano Lett. 10, 3460–3462. (doi:10.1021/nl9016623) 10.1021/nl9016623 19645460

[42] Ham HT, Choi YS, Chung IJ 2005 An explanation of dispersion states of single-walled carbon nanotubes in solvents and aqueous surfactant solutions using solubility parameters. J. Colloid Interface Sci. 286, 216–223. (doi:10.1016/j.jcis.2005.01.002) 15848419 10.1016/j.jcis.2005.01.002

[43] Inam F, Yan H, Reece MJ, Peijs T 2008 Dimethylformamide: an effective dispersant for making ceramic-carbon nanotube composites. Nanotechnology 19, 195710 (doi:10.1088/0957-4484/19/19/195710) 21825728 10.1088/0957-4484/19/19/195710

[44] Song SH, Park KH, Kim BH, Choi YW, Jun GH, Lee DJ, Kong BS, Paik KW, Jeon S 2013 Enhanced thermal conductivity of epoxy–graphene composites by using non-oxidized graphene flakes with non-covalent functionalization. Adv. Mater. 25, 732–737. (doi:10.1002/adma.201202736) 23161437 10.1002/adma.201202736

[45] Naebe M, Wang J, Amini A, Khayyam H, Hameed N, Li LH, Chen Y, Fox B 2014 Mechanical property and structure of covalent functionalised graphene/epoxy nanocomposites. Sci. Rep. 4, 4375 (doi:10.1038/srep04375) 24625497 10.1038/srep04375 PMC3953726

[46] Dong X, Chen J, Ma Y, Wang J, Chan-Park MB, Liu X, Wang L, Huang W, Chen P 2012 Superhydrophobic and superoleophilic hybrid foam of graphene and carbon nanotube for selective removal of oils or organic solvents from the surface of water. Chem. Commun. 48, 10 660–10 662. (doi:10.1039/C2CC35844A) 10.1039/c2cc35844a 23001335

[47] Zhang YH, Zhou KG, Xie KF, Zhang HL, Peng Y, Wang CW 2012 Tuning the magnetic and transport properties of metal adsorbed graphene by co-adsorption with 1, 2-dichlorobenzene. Phys. Chem. Chem. Phys. 33, 11 626–11 632. (doi:10.1039/C2CP41370A) 10.1039/c2cp41370a 22820954

[48] Li X, Chen G 2009 Surface modified graphite nanosheets used as adsorbent to remove 1,2-dichlorobenzene from water. Mater. Lett. 11, 930–932. (doi:10.1016/j.matlet.2009.01.042)

[49] Zhou X, Yang X 2012 Improved dispersibility of graphene oxide in o-dichlorobenzene by adding a poly(3-alkylthiophene). Carbon 50, 4566–4572. (doi:10.1016/j.carbon.2012.05.041)

[50] Ramanathan T et al. 2008 Functionalized graphene sheets for polymer nanocomposites. Nat. Nanotechnol. 3, 327–331. (doi:10.1038/nnano.2008.96) 18654541 10.1038/nnano.2008.96

[51] Liu Q, Liu Z, Zhang X, Yang L, Zhang N, Pan G, Yin S, Chen Y, Wei J 2009 Polymer photovoltaic cells based on solution-processable graphene and P3HT. Adv. Funct. Mater. 19, 894–904. (doi:10.1002/adfm.200800954)

[52] Moon IK, Lee J, Ruoff RS, Lee H 2010 Reduced graphene oxide by chemical graphitization. Nat. Commun. 1, 73 (doi:10.1038/ncomms1067) 20865806 10.1038/ncomms1067

[53] Liu LH, Lerner MM, Yan M 2010 Derivitization of pristine graphene with well-defined chemical functionalities. Nano Lett. 10, 3754–3756. (doi:10.1021/nl1024744) 20690657 10.1021/nl1024744 PMC2940829

[54] Qiu SL, Wang CS, Wang YT, Liu CG, Chen XY, Xie HF, Huang YA, Cheng RS 2011 Effects of graphene oxides on the cure behaviors of a tetrafunctional epoxy resin. Express Polym. Lett. 5, 809–818. (doi:10.3144/expresspolymlett.2011.79)

[55] Wang X, Jin J, Song M 2013 An investigation of the mechanism of graphene toughening epoxy. Carbon 65, 324–333. (doi:10.1016/j.carbon.2013.08.032)

[56] Atif R, Inam F 2016 Reasons and remedies for the agglomeration of multilayered graphene and carbon nanotubes in polymers. Beilstein J. Nanotechnol. 7, 1174–1196. (doi:10.3762/bjnano.7.109) 27826492 10.3762/bjnano.7.109 PMC5082316

[57] Gordana M, Visakh PM. 2016 Rubber nano blends: preparation, characterization and applications, 1st edn Basel, Switzerland: Springer.

[58] Saleem H, Edathil A, Ncube T, Pokhrel J, Khoori S, Abraham A, Mittal V 2006 Mechanical and thermal properties of thermoset-graphene nanocomposites. Macromol. Mater. Eng. 301, 231–259. (doi:10.1002/mame.201500335)

